# Fine-Needle Aspiration Biopsy as a Preoperative Procedure in Patients with Malignancy in Solitary and Multiple Thyroid Nodules

**DOI:** 10.1371/journal.pone.0146883

**Published:** 2016-01-19

**Authors:** Krzysztof Kaliszewski, Dorota Diakowska, Beata Wojtczak, Marta Strutyńska-Karpińska, Paweł Domosławski, Krzysztof Sutkowski, Mateusz Głód, Waldemar Balcerzak, Zdzisław Forkasiewicz, Tadeusz Łukieńczuk

**Affiliations:** 1 1st Department and Clinic of General, Gastroenterological and Endocrine Surgery, Wroclaw Medical University, Wroclaw, Poland; 2 Department and Clinic of Gastrointestinal and General Surgery, Wroclaw Medical University, Wroclaw, Poland; University of Utah Health Sciences Center and ARUP Laboratories, UNITED STATES

## Abstract

**Background:**

Fine-needle aspiration biopsy (FNAB) is a recognized technique for the basic, preoperative cytological diagnosis of thyroid nodules.

**Aim of the Study:**

To analyze the accuracy of FNAB in the diagnosis of thyroid cancer in patients with solitary and multiple thyroid nodules and to compare the demographic, clinical and pathological characteristics of patients with thyroid carcinoma in solitary and multiple tumors.

**Materials and Methods:**

The case records of 2,403 patients with solitary and multiple thyroid tumors treated consecutively between 2008 and 2013 were analyzed retrospectively. We selected 1,645 for further analysis. A solitary thyroid nodule was observed in 493 patients, and multiple nodules were detected in 1,152 patients. Further classification of the patients in these two groups was performed on the basis of the FNAB results, type of surgery performed and histopathology. TC was histopathologically confirmed in 166 patients, and benign disease was found in 1,479. The TC patients were assigned to the study group, and those with benign thyroid disease were placed into the control group. The study group was divided into two subgroups according to the presence of cancer in a single thyroid nodule or in multiple nodules. Malignancy in a solitary thyroid nodule was diagnosed in 98 (59.0%) patients, and cancer in multiple nodules was diagnosed in 68 (41.0%). Comparative analyses of the demographic, clinical and histopathological characteristics were performed for both subgroups. The following statistical analyses were performed: comparative characteristic of subgroups, ROC analysis for study group and subgroup of patients, and multivariable logistic regression analysis for study group.

**Results:**

The rate of prediction of TC by FNAB was three times higher in the patients with a solitary thyroid nodule compared with those with multiple thyroid nodules and it was statistically significant (p<0.001). The rate of total thyroid resection and lack of necessity for reoperation were also significantly higher in the TC patients with a solitary nodule. The histopathological results showed that significantly more patients with a solitary nodule had advanced-stage TC (stage III or IV) and tumor progression (pT3 or pT4) (p = 0.002 for both). ROC analysis demonstrated that the overall accuracy of FNAB as a predictor of thyroid cancer presence was high, especially for the subgroup of patients with a solitary thyroid nodule (AUC = 0.958, p<0.0001). Multivariable logistic regression analysis confirmed that a positive FNAB result was the sole predictor of the performance of total resection in the TC study group (p<0.0001), while a negative FNAB result and the presence of a papillary cancer type were independent predictors of the risk of reoperation (p<0.0001 and p = 0.002, respectively).

**Conclusions:**

FNAB often produces false-negative results in patients with multiple malignant thyroid tumors, which results in reoperation in many cases. False-negative FNAB results are rare in patients with a solitary tumor. Because of the low predictive capacity of FNAB for thyroid cancer in patients with multiple thyroid tumors, total thyroid excision should be considered in most cases despite a "negative" (no malignant) FNAB result.

## Introduction

The value of fine-needle aspiration biopsy (FNAB) for diagnosis of thyroid pathologies has been established; however, in some specific situations, its reliability is debatable. This diagnostic tool is appropriate and valuable for the evaluation of single thyroid tumors, but has shown less efficacy for that of multinodular thyroid glands.

A false-negative FNAB result (when the appearance of cancer in a multinodular goiter is missed) is a relatively rare occurrence in diagnosis of thyroid pathologies; however, it may occur in some cases of multinodularity of the thyroid gland. This result can arise when FNAB is performed on a targeted benign nodule, and a malignant process is recognized postoperatively in another nodule. Alternatively, certain technical limitations, such as collection of nondiagnostic samples, may cause a false-negative FNAB result. Currently, fine-needle aspiration cytology (FNAC) is one of the most popular and accurate techniques for presurgically evaluating thyroid nodules [1]. It is possible to plan the extent of surgery and further treatment based on the results of this evaluation. The most valuable information that FNAC provides is whether a nodule has neoplastic potential.

The prevalence of multinodular goiter is very high worldwide, particularly in endemic areas [2–5]. Approximately 93% of all thyroid nodules are benign. Papillary thyroid carcinoma is the most common thyroid malignancy and sometimes can present with a very aggressive course.

Despite enormous progress in clinical diagnostics in the last years, both the diagnosis and treatment of thyroid cancer remain challenging. An estimated diagnosis based on FNAB findings is not always concordant with that based on the final histopathological examination results. Additionally, incidental thyroid malignancy in a multinodular goiter is a very similar clinical situation that can occur and may interfere with diagnosis. One of the main difficulties of management of suspected malignancy in multiple thyroid tumors involves the decision of which nodule should be biopsied. There is no agreement among specialists regarding the selection of a nodule for biopsy, but sonography characteristics that are predictive of malignancy have been described. They include a single nodule, a solid lesion, hypoechogenicity and an irregular shape, edge and size [6–13]. Some authors have indicated that thyroid nodules with specific sonography characteristics should be biopsied; however, others believe that there are no sonographic features unique to benign or malignant lesions and that all nodules should be biopsied [10, 14].

Thyroid tumors are very frequently observed in middle-aged and older patients in endemic areas [15]. Analysis of autopsy surveys has revealed that their prevalence is up to 30% in high-risk groups [16]. Some authors have suggested that the rate of detection of incidental thyroid carcinoma after surgery in patients with apparently benign multinodular goiter (MNG) ranges from 3–16.6% [17]. Additionally, they have noted that one-third of all patients who have received subtotal thyroidectomy for MNG require further surgical treatment [17].

Patients with a single or dominant nodule have been reported to be at a higher risk of malignancy than those with multiple nodules [18]. This finding might prompt the choice of less radical surgery in the case of a multinodular thyroid gland. However, some authors have indicated that a nodule in a multinodular gland that has grown progressively, has become definitely dominant, and has changed in consistency (features that might be observed during ultrasonography) carries the same risk of malignancy as a solitary nodule [18].

FNAB should improve the efficacy of the diagnostic evaluation of thyroid nodules, and it should not lead to unjustified increases in improper qualification and unnecessary surgical procedures. On the other hand, when a "negative result" (no suspicion of malignancy) for FNAB prompts the choice of less radical surgery in the case of malignancy, reoperation may be needed. Thus, diagnostic guidelines should be used in routine, clinical evaluations to improve the efficacy of thyroid tumor management. However, in some cases, these guidelines require individual, pragmatic and practical considerations.

The aim of the present study was to analyze the accuracy of FNAB in diagnosis of thyroid cancer in patients with solitary and multiple thyroid nodules and to compare the demographic, clinical and pathological characteristics of patients with cancer in solitary and multiple thyroid nodules.

## Materials and Methods

Our study protocol was approved by the Bioethics Committee of Wroclaw Medical University (signature number: KB 272/2015). We obtained oral consent from the participants instead of written consent because the data were analyzed anonymously.

### Study group

The case records of 2,403 patients with solitary and multiple thyroid nodules who were treated consecutively between January 2008 and March 2013 at the Department of General, Gastroenterological and Endocrine Surgery of Wroclaw Medical University (Poland) were analyzed retrospectively. The presence of solitary or multiple thyroid nodules, observed by physical examination and imaging techniques, such as ultrasonography, computed tomography, scintigraphy and magnetic resonance, was a criterion for FNAB. All of the patients underwent this procedure.

**Fig 1:**
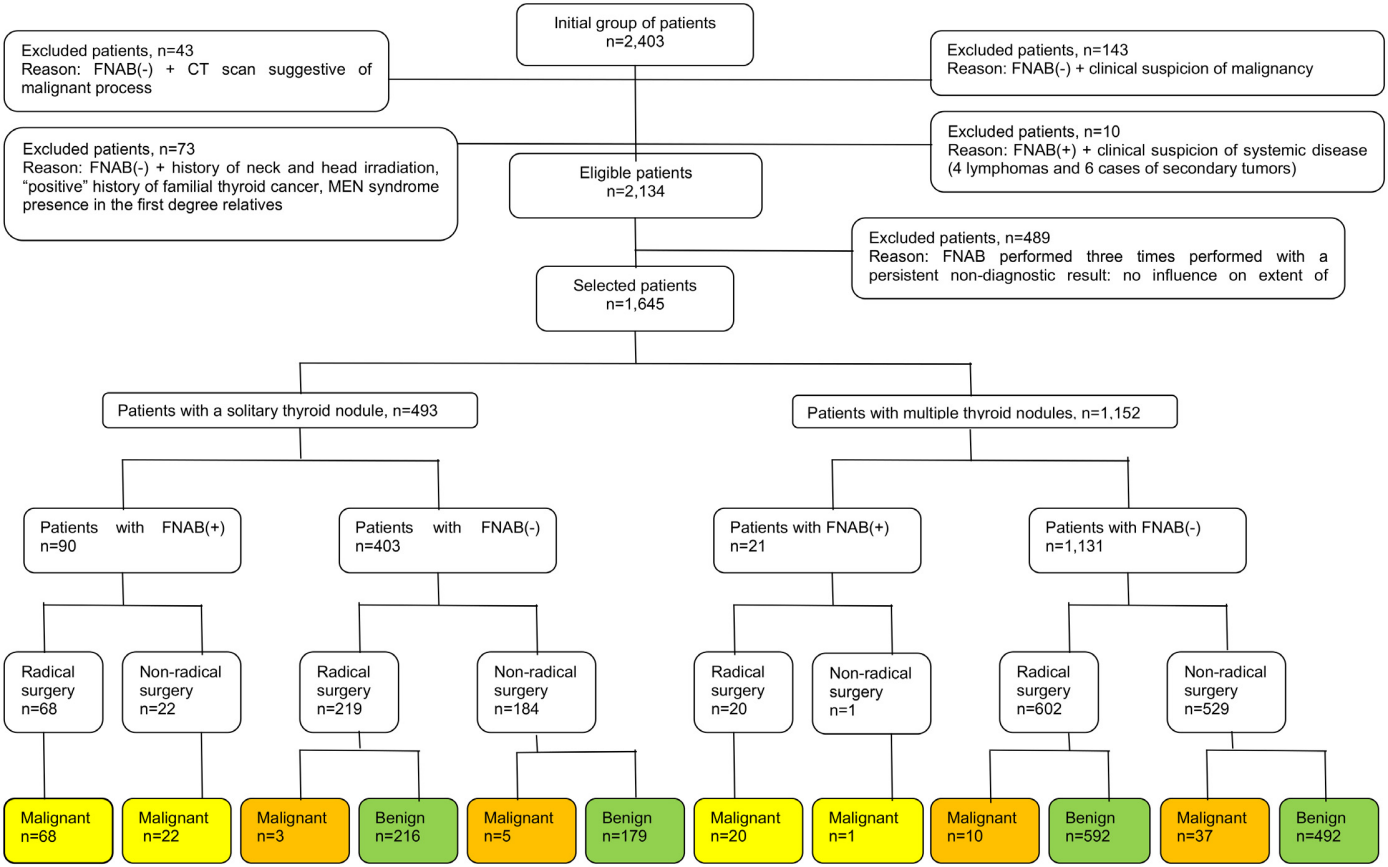
Selection of study group from 2,403 individuals referred for surgery from 2008 to 2013. All participants underwent FNAB. FNAB: fine-needle aspiration biopsy, CT: computed tomography, MEN: multiple endocrine neoplasm. FNAB(-): “negative” fine-needle aspiration biopsy result, no suspicion of malignancy; FNAB(+): “positive” fine-needle aspiration biopsy result, suspicion of malignancy; (*): patients with FNAB(-) and benign histopathology result, n = 1,479; (**): patients with FNAB(-) and malignant histopathology result, n = 55; (***): patients with FNAB(+) and malignant histopathology result, n = 111.

The steps for patient selection are presented in Fig 1. From the initial group of patients (n = 2,403) we excluded those whose computed tomography (CT) findings were suggestive of a malignant process (infiltration of the trachea) (n = 43), those with clinical suspicion of malignancy (hoarseness, dysphagia, enlarged and pathologic lymph nodes of the neck) (n = 143), clinical suspicion of systemic disease (lymphoma or metastatic tumor) (n = 10), a history of head and neck irradiation, a "positive" history of familial thyroid cancer, and the presence of multiple endocrine neoplasm (MEN) syndrome in first-degree relatives (n = 73) to obtain a homogenous group of patients (n = 2,134) with the pathology localized only within the thyroid gland and without other factors that would influence the final decision of the extent of surgery (radical vs. non-radical). Next, from this group of patients (eligible patients, n = 2,134), 489 were excluded from the study because of the persistent non-diagnostic FNAB results. After this selection process, 1,645 patients remained who comprised the main group of examined patients. Among these patients, a solitary thyroid nodule was observed in 493, and multiple thyroid nodules were detected in 1,152. Further classification of the patients in each of these two groups was performed on the basis of the FNAB results (FNAB(+) "positive"—malignancy suspicion or FNAB(-) "negative"—no malignancy suspicion), the receipt of radical/non-radical surgery and the histopathology results. Finally, thyroid cancer was histopathologically confirmed in 166 patients, and benign disease of the thyroid was found in 1,479. The patients with TC were assigned to the study group, and those with benign thyroid disease were placed in the control group. The average age of the patients with thyroid cancer patients was 53±16 years, and that of those with benign thyroid disease was 48±19 years (p>0.05). There was no significant difference in the gender distribution between the patients with TC and those without cancer (female/male: 142/24 for the TC patients and 1,168/311 for those without cancer, p>0.05). Next, the study group was divided into two subgroups according to the presence of cancer in a single thyroid nodule or in multiple nodules. Malignancy in a solitary thyroid nodule was diagnosed in 98 patients (59.0%), and cancer in multiple nodules was diagnosed in 68 (41.0%). Comparative analyses of the demographic, clinical and histopathological characteristics (according to pTNM staging, as described in the 7^th^ edition of the AJCC Cancer Staging Manual) were performed for both subgroups.

### Statistical analysis

Numbers and percentages were calculated for qualitative variables, and the mean and standard deviation were calculated for quantitative variables. The distribution of the data was tested using the Shapiro-Wilk normality test.

Differences between the two subgroups of TC patients were analyzed by the chi-square test with Fisher’s exact test or by Student’s t test.

The diagnostic potential of FNAB was determined by receiver operating characteristic (ROC) analysis and was expressed in terms of the area under the ROC curve (AUC). The accuracy, sensitivity, specificity, positive predictive value (PPV), negative predictive value (NPV), likelihood ratio of positive results (LR(+)), and likelihood ratio of negative results (LR(-)) were also calculated.

The stepwise method of multiple logistic regression was used for the determination of independent predictors that influenced the decision for radical surgery and reoperation p>0.1 as an exclusion criterion in univariable analysis) for the study group (n = 166). Odds ratios and 95% confidence intervals (± 95%CI) were also calculated.

A two-tailed p-value of <0.05 was considered statistically significant. Statistical analyses were performed using STATISTICA 10.0 software (StatSoft Inc., Tulsa, OK, USA) and MedCalc Statistical Software v. 13.2.2 (MedCalc Software, Ostend, Belgium).

## Results

### Demographic, clinical and pathological characteristics of patients with cancer in solitary or multiple thyroid nodules

The characteristic of the patients in this study group and the comparative characteristics of the subgroups of patients with cancer in solitary and multiple thyroid nodules are shown in Table 1. There were no significant differences in age, gender or cancer type between the two subgroups of cancer patients. However, the rate of prediction of TC by FNAB was three times higher in the patients with cancer in a solitary thyroid nodule compared with those with cancer in multiple nodules, and this difference was statistically significant (p<0.001). The rate of radical thyroid resection and lack of necessity for reoperation were also significantly increased in the patients with malignancy in a solitary nodule compared with those with cancer in multiple nodules (p = 0.0002 and p<0.001, respectively). The histopathological results showed that significantly more patients with advanced disease (stage III or IV) and tumor progression (pT3 or pT4) had TC in a solitary thyroid nodule (p = 0.002 for both).

**Table 1:** Demographic, clinical and pathological characteristics of TC patients. Descriptive data are presented as numbers (n) and percentages (%) or as the mean ± standard deviation (± SD).

Parameter	A: Total TC patients (n = 166)	B: Patients with TC in solitary thyroid nodule (n = 98)	C: Patients with TC in multiple thyroid nodules (n = 68)	Chi^2^ test (B *vs* C)	p-value (B *vs* C)
Age (years)	53.0±16.4	53.4±17.7	52.5±14.3	t = 0.37	0.710
Age:				0.51	0.473
<45	49 (29.5)	31 (31.6)	18 (26.5)		
≥45	117 (70.5)	67 (68.4)	50 (73.5)		
Gender:				0.006	0.939
female	142 (85.5)	84 (85.7)	58 (85.3)		
male	24 (14.5)	14 (14.3)	10 (14.7)		
FNAB:				67.3	<0.001*
FNAB(+)	111 (66.9)	90 (91.8)	21 (30.9)		
FNAB(-)	55 (33.1)	8 (8.2)	47 (69.1)		
Type of operation:				13.5	0.0002*
radical	101 (60.8)	71 (72.5)	30 (44.1)		
non-radical	65 (39.2)	27 (27.5)	38 (55.9)		
Reoperation				23.5	<0.001*
no	55 (33.1)	80 (81.6)	31 (45.6)		
yes	111 (66.9)	18 (18.4)	37 (54.4)		
Type of cancer:				6.6	0.159
papillary	138 (83.1)	77 (78.6)	61 (91.0)		
follicular	12 (7.2)	8 (8.2)	4 (6.0)		
medullary	5 (3.0)	5 (5.1)	0 (0.0)		
undifferentiated	8 (4.8)	6 (6.1)	2 (3.0)		
sarcoma	2 (1.2)	2 (2.0)	0 (0.0)		
pTNM stage:				9.6	0.022*
I	105 (63.3)	54 (55.1)	51 (75.0)		
II	25 (15.1)	15 (15.3)	10 (14.7)		
III	17 (10.2)	13 (13.3)	4 (5.9)		
IV	19 (11.4)	16 (16.3)	3 (4.4)		
pTNM:				8.8	0.002*
I+II	130 (78.3)	69 (70.4)	61 (89.7)		
III+IV	36 (21.7)	29 (29.6)	7 (10.3)		
pT:				9.6	0.023*
pT1	101 (60.8)	53 (54.1)	48 (70.6)		
pT2	26 (15.7)	14 (14.3)	12 (17.7)		
pT3	18 (10.8)	13 (13.3)	5 (7.4)		
pT4	21 (12.7)	18 (18.4)	3 (4.4)		
pT:				8.8	0.002*
pT1+pT2	127 (76.5)	67 (68.4)	60 (88.2)		
pT3+pT4	39 (23.5)	31 (31.6)	8 (11.8)		
pN:				26.2	<0.001*
pN0	69 (41.6)	50 (51.0)	19 (27.9)		
pN1a	18 (10.8)	15 (15.3)	3 (4.4)		
pN1b	10 (6.0)	8 (8.2)	2 (2.9)		
pNx	69 (41.6)	25 (25.5)	44 (64.7)		
pM:				28.5	<0.001*
pM0	91 (54.8)	68 (69.4)	23 (33.8)		
pM1	5 (3.0)	5 (5.1)	0 (0.0)		
pMx	70 (42.2)	25 (25.5)	45 (66.2)		

*: statistically significant

FNAB: fine-needle aspiration biopsy

### Diagnostic potential of FNAB in patients with solitary and multiple thyroid nodules

The diagnostic potential of FNAB was evaluated in terms of the capacity to rule out thyroid malignancy in subjects with benign thyroid disease (the controls). The overall accuracy of FNAB as a predictor of the presence of thyroid cancer was high, especially in the patients with a solitary thyroid nodule (Table 2). The sensitivity of FNAB was the lowest (30%) and the specificity was the highest (100%) in the patients with TC in multiple nodules.

**Table 2:** Diagnostic potential of FNAB as indicator of thyroid cancer presence.

	FNAB in selected patients (n = 1,645)	FNAB in patients with solitary thyroid nodule (n = 473)	FNAB in patients with multiple thyroid nodules (n = 1,152)
AUC (95%CI)	0.829 (0.784–0.874)	0.958 (0.926–0.991)	0.647 (0.567–0.727)
p-value	<0.0001	<0.0001	<0.0001
SE	0.023	0.017	0.041
sensitivity	0.66	0.92	0.30
specificity	1.00	1.00	1.00
accuracy	0.97	0.98	0.96
LR(+)	1.00	1.00	1.00
LR(-)	0.34	0.08	0.71
PPV	1.00	1.00	1.00
NPV	0.96	0.98	0.96

AUC: area under ROC curve, 95%CI: 95% confidence interval, SE: standard error, LR(+): likelihood ratio of positive results, LR(-): likelihood ratio of negative results, PPV: positive predictive value, NPV: negative predictive value

### Role of FNAB procedure and clinico-pathological characteristics as independent predictors of the decision for radical operation and reoperation in TC patients

Multiple logistic regression analyses were performed for all TC patients in the study group (n = 166).

Univariable analysis revealed that a positive FNAB result and the presence of a solitary thyroid nodule were independent factors related to the performance of radical resection in the TC patients (Table 3). However, multivariable analysis confirmed that a positive FNAB result was a sole predictor of the performance of total resection in the TC patients (p<0.0001).

**Table 3:** Multiple univariable and multivariable logistic regression analyses of prediction of radical thyroid resection (radical/non-radical; 1/0) in TC patients.

	univariable analysis			multivariable analysis		
	OR	95% CI	p-value	OR	95% CI	p-value
Age: <45 vs ≥45	0.86	0.43–1.73	0.679	-	-	-
Gender: for “female”	0.75	0.30–1.87	0.528	-	-	-
FNAB: for FNAB(+)	12.36	5.67–26.93	<0.0001*	19.45	6.05–62.5	<0.0001*
Thyroid nodules: for solitary	3.33	1.73–6.43	0.0003*	1.89	0.61–5.88	0.270

OR: odds ratio, 95%CI: 95% confidence interval

*: statistically significant

The TC patients with multiple thyroid nodules, in which cancer was not confirmed by FNAB or the presence of papillary TC, demonstrated a significantly increased risk of reoperation, (p<0.05 for these three parameters) (Table 4). Multivariable analysis indicated that only a negative FNAB result and the presence of a papillary cancer type were independent predictors of the risk of reoperation (p<0.0001 and p = 0.002, respectively).

**Table 4:** Multiple logistic regression analysis of prediction of reoperation (yes/no; 1/0) in TC patients.

	univariable analysis			multivariable analysis		
	OR	95% CI	p-value	OR	95% CI	p-value
Age: <45 vs. ≥45	0.85	0.41–1.75	0.655	-	-	-
Gender: male vs female	0.81	0.31–2.09	0.655	-	-	-
FNAB: for FNAB(-)	4.29	2.85–6.48	<0.0001*	5.02	2.69–9.33	<0.0001*
Thyroid nodule: for multiple	5.30	2.62–10.73	<0.0001*	1.02	0.029–3.59	0.974
Histological type of cancer: for papillary	0.41	0.18–0.94	0.034*	0.18	0.06–0.55	0.002*
pTNM: I+II vs III+IV	1.22	0.52–2.89	0.647	-	-	-
pT: 1+2 *vs* 3+4	1.25	0.54–2.89	0.593	-	-	-
pN: 0 *vs* 1 and other	1.6	0.35–7.36	0.540	-	-	-
pM: 0 *vs* 1	2.96	0.28–13.19	0.359	-	-	-

## Discussion

A large number of studies have demonstrated the high overall accuracy of FNAB for evaluation of thyroid nodules, and this finding has been confirmed, particularly for patients with a single nodule and those with some malignant characteristics observed on ultrasonography [18]; however, FNAB has shown less accuracy in the evaluation of multinodular thyroid glands. In addition, previous studies have reported that the accuracy of FNAB is lower for multiple nodules than for a single nodule [18, 19], in agreement with the results of our study. We found that the rate of prediction of TC by FNAB in the patients with a solitary thyroid nodule was three times higher than that in patients with multiple nodules. In our opinion, patient qualification for the FNAB procedure should be determined very carefully. In the case of a negative FNAB result, the surgeon may perform a less radical procedure, even if the patient presents with some unfavorable features and the final histopathological examination may reveal that the tumor is malignant. The completion of surgery might be required in some of these cases. A false-negative FNAB result may cause patients and physicians to avoid surgery, even if some other characteristics indicate suitability for surgery. Another very important issue is the overestimation of false-negative results, which can occur in the case of multinodularity of the thyroid gland. This situation is possible with multiple tumors, in which a “negative” nodule sampled by FNAB and a “positive” nodule recognized by histopathological examination are not the same nodules. The question then arises of whether the risk of malignancy of a thyroid nodule is higher for nondiagnostic FNAB results. Thus, the next step in the diagnostic evaluation of thyroid nodules should be taken after several nondiagnostic samples are obtained. Other questions include whether the patient qualifies for surgery in such a situation and whether the next biopsy procedure should be repeated, and, if so, the number of times that it should be repeated. These questions have been posed by Coorough et al. [19], who reviewed the reports of 4,286 patients with thyroid nodules who underwent FNAB. Nondiagnostic results were obtained for 259 (6%) of the patients. Among them, 62 (24%) underwent thyroidectomy, and 74 (29%) received a repeat FNAB, and cytologic or histopathologic diagnosis was achieved in 136 (53%) of the patients. This group noted that the incidence of malignancy in the patients with a previous nondiagnostic FNAB was significantly higher. In our study, we repeated FNAB two to three times after obtaining nondiagnostic results. In 489 cases, we obtained non-diagnostic samples during these three repeated procedures, so they were excluded.

Interesting observations have been reported by Ravetto et al. [20], who suggested that the probability of malignancy in a thyroid nodule is significantly reduced after a "negative" (no cancer suspicion) FNAB result. This group has confirmed that the risk of malignancy in such cases ranges from 0.4% to 4%. Ravetto preferred to follow these patients every 2 years and to perform a repeat fine-needle aspiration biopsy after 4–5 years. The indolent course of thyroid cancer in some cases justifies a long interval between clinical follow-up examinations, as noted by Mazzaferri et al. [21]. Based on the available literature concerning this issue, the thyroid nodules most often qualifying for FNAB are those with 131 J uptake of less than or equal to that of the normal surrounding tissues and other surrounding nodules. Thus, all cold and warm (indeterminate) nodules are biopsied one or more times [20]. In Ravetto et al.’s study, autonomous nodules were biopsied in 2.29% of the patients, although we agree with the opinion of some authors concerning the disqualification of hot (autonomous) nodules for FNAB. These nodules are very exceptionally malignant, and they need careful monitoring [20].

Some authors have suggested that the diagnostic limitations of FNAB, for example, the indeterminate category of samples, may be partially overcome by performing molecular analysis [22]. They have added that although the impact of detecting point mutations and rearrangements in fine-needle aspirations (FNAs) has most likely been overestimated in previous studies, molecular FNA analysis improves presurgical diagnosis. In their opinion, detection of a BRAF mutation in FNAs can improve the selection of the surgical procedure and decision for the extent of surgery [22]. Some authors have indicated that the contribution of molecular analysis of FNAC will be much more significant in the future because the cytologic findings will be reevaluated in some cases, and the potential diagnostic contribution of the available data will be considered [23].

FNAB is potentially a very accurate diagnostic procedure, particularly when it is performed by experienced physicians. However, even the most accurate diagnostic tool should be used rationally to achieve the largest practical gain. Until new genetic or molecular tests are identified that are diagnostically valuable for malignancy detection, clinical information and diagnostic findings remain very helpful for identifying patients with multifocal tumors who are at an increased risk of malignancy. We suggest that FNAB results should be considered together with individual patients’ clinical characteristics to be of the most value. The time has passed for the use of FNAB as the sole triage tool for evaluation of thyroid tumors without consideration of any information on their malignant potential [24–26]. In almost all cases, some information should be considered before making a decision concerning the extent of surgery in patients with a multinodular goiter, such as demographic data, lesion size, scintigraphy findings and patient age. Of course, this information may not always be useful. In our study, there were no significant differences in age, gender or cancer type between the cancer patients with solitary and multiple thyroid nodules. Ersoz et al. have suggested that all patients older than 37 years of age require careful investigation. They have stated that the presence of thyroid nodules of greater than pT1b in size might be indicative of malignancy [23]. Additionally, some authors have observed a female predominance among patients with neoplastic nodules and among those with thyroid malignancy [23, 27]; however, other authors have contradicted these findings [28].

In our study, the histopathological results showed that significantly more of the patients with a solitary thyroid nodule had advanced-stage TC (stage III or IV) and tumor progression (pT3 or pT4). Lymph node and distant metastases were not definite in 25% of the patients with cancer in a solitary thyroid nodule and in approximately 65% of those with malignancy in multiple nodules. Therefore, the finding of the significantly higher number of stages pN1 and pM1 tumors in the patients with cancer in a solitary thyroid nodule should be interpreted carefully.

Because of the differences in the prediction of TC by FNAB between the patients with a solitary thyroid nodule and those with multiple thyroid nodules, these two groups were analyzed independently. In both groups, significant differences in the choice of surgical procedure and the necessity for reoperation were observed between the cancers confirmed and not confirmed by FNAB (p<0.05 for both parameters for a solitary thyroid nodule; and p<0.0001 for both parameters for multiple thyroid nodules). In our opinion, total thyroidectomy might have been the appropriate surgical option for almost all of the patients included in our study with multiple thyroid tumors because almost 70% of them had presumably benign tumors. Many investigators believe that total thyroidectomy is safe, effective and the recommended treatment for this pathology at advanced thyroid surgery centers, and its use has corresponded with a decrease in secondary thyroidectomy for incidental cancer or recurrent goiter [29–32]. In our analysis, sixteen patients were over 60 years of age, and this finding might be in support of the argument for total thyroidectomy because, as Puzziello et al. have suggested, age is an independent prognostic factor for cancer, and it is not a contraindication for total thyroidectomy [33]. Thus, in patients older than 60 years of age with multifocal thyroid tumors, this radical surgical procedure can be performed despite a negative FNAB result for malignancy. Boger et al. [34] have described additional clinical characteristics that could be independent prognostic factors for total thyroidectomy in patients with multiple thyroid nodules despite a benign FNAB result, including preoperative diagnosis of a large solitary or dominant nodule, intrathoracic and nontoxic multinodular goiter and mental disability, which may impair the accuracy of long-term observation. Some authors have suggested that the nodule dimension strongly influences the reliability of FNAB results. Ucar et al. [35] have reported that the false-negative rate of FNAB is higher for the larger nodules than for minor ones. They have concluded that this diagnostic evaluation is significant in the case of positive FNAB results for malignancy; however, it is not very reliable in the case of negative (benign) results. Another argument supporting total radical surgery that can be considered for all patients with multiple thyroid tumors without suspicion of malignancy is that they may undergo the surgery at a high-volume surgical center. Some authors have suggested that the complication rate of total surgery is the same as that of partial thyroidectomy when surgery is performed at a high-volume surgical center [36, 37]. We and others believe that total thyroidectomy performed by expert surgeons with experience in thyroid surgery and total thyroidectomy to treat patients with multinodular goiter should be considered safe and effective [17]. The medical center where the authors of this study are employed is a surgical reference center with experience in thyroid surgery. Thus, we could extend the indication for more radical surgery to include most of our patients with multifocal thyroid lesions, particularly those with incidental thyroid cancer discovered postsurgically in a multinodular goiter that would not have been reoperable if it had been detected before the initial surgery.

FNAB typically produces false-negative results in patients with malignancy in multiple thyroid tumors. In our study, the percentage of false negatives reached 69%, necessitating reoperation in many cases (54% of our patients). False-negative FNAB results are not very common in individuals with a solitary tumor, accounting for 8% of our patients. Because of the low predictive capacity of FNAB for thyroid cancer in patients with multiple thyroid tumors, total thyroid excision should be considered in most cases, despite a "negative" (no malignancy) FNAB result.

## Supporting Information

S1 Dataset Data used in analyses. (XLS)
